# A comparison in tracheostomy weaning and complications rate between neuro critical care patients and general critical care patients

**DOI:** 10.1186/2197-425X-3-S1-A942

**Published:** 2015-10-01

**Authors:** M Colomo, T Chamroo, N Muchoki, J Darroch, G De la Cerda

**Affiliations:** Intensive Care Unit, Queen's Hospital, Romford, United Kingdom

## Introduction

Tracheostomies are the most common procedure performed in the intensive care units for patients who need prolonged respiratory support (>21 days).

## Objectives

We aimed to compare the differences in the tracheostomy weaning and the complications rate between neuro critical care and general critical care patients.

## Methods

Retrospective cohort analysis of prospective collected data at a tertiary neuroscience centre. All the patients requiring tracheostomy in our Intensive Care Department from October 2012 to March 2015 were included. The patients were allocated in 2 different units according to the reason for admission (Neurocritical Care Unit and General Intensive Care Unit). Tracheostomy weaning was performed in accordance with local protocols. Demographics, tracheostomy data (timing, reason, technique), complications during weaning from mechanical ventilation and timing of decannulation were collected.

## Results

A total of 165 patients underwent tracheostomies during ICU stay. Length of weaning from mechanical ventilation post tracheostomy was longer in General ICU patients than in Neuro ICU patients (median 6 days, Q1-Q3 4, 10 days vs median 3 days, Q1-Q3 2, 8 days). However, length of decannulation was longer in Neuro ICU patients (median 21 days, Q1-Q3 13,29.5 days vs median 18 days, Q1-Q3 11.5, 33 days). In both cases this difference was not statistically significant (p = 0,06 for weaning and p = 0,79 for decannulation). Regarding the complications during the weaning process, they were more frequent in Neuro ICU patients than in General ICU patients (56,8% vs 33,7%). In Neuro ICU patients the most common complication that could delay the weaning was the low level of consciousness (28 patients) following by the inability to manage the oral secretions and the lack of airway reflexes (11 and 6 patients). Chest infections, poor cough and low level of consciousness were observed more frequently in General ICU patients as a complications during the weaning (9, 5 and 5 patients respectively).

## Conclusions

1. We have not found statistically significant differences in the length of weaning and decannulation after tracheostomy between neuro and general intensive care patients.

2. The pattern of complications that can delay the weaning and decannulation process is different according to the reason for admission in Critical Care.

**Table 1:** Table 1

	Neuro Critical Care Unit	General Critical Care Unit
Total number of patients	88	77
Age (years)	54,4 ± 16,8	60,9 ± 16,9
Gender (M/F %)	55/33 %	61/39 %
Technique percutaneous/surgical %	56/32 %	70/30%

*[Demographics]*

**Figure 1:**
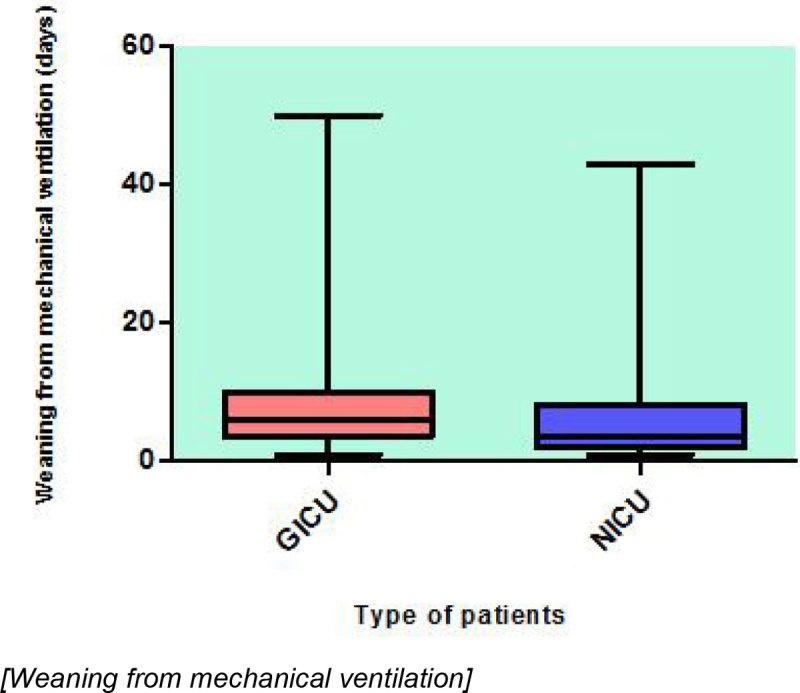
**Weaning from mechanical ventilation**.

**Figure 2:**
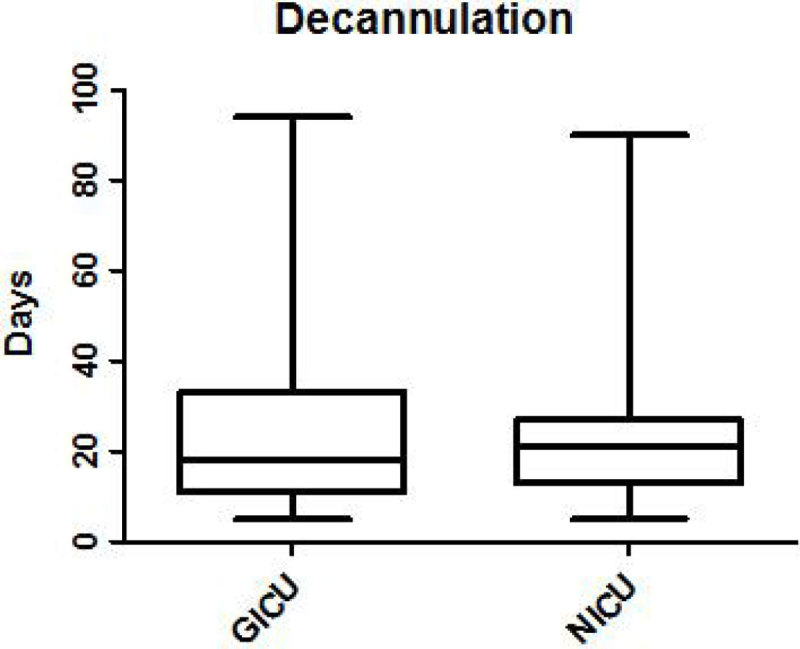
**Decannulation**.
